# Discourse Context Immediately Overrides Gender Stereotypes during Discourse Reading: Evidence from ERPs

**DOI:** 10.3390/brainsci13030387

**Published:** 2023-02-23

**Authors:** Yanan Du, Yaxu Zhang

**Affiliations:** 1 School of Psychological and Cognitive Sciences, Beijing Key Laboratory of Behavior and Mental Health, Peking University, Beijing 100871, China; 2 Key Laboratory of Machine Perception (Ministry of Education), Peking University, Beijing 100871, China

**Keywords:** gender stereotypes, pragmatics, discourse context, language comprehension, event-related brain potentials

## Abstract

This study investigated how local gender stereotype information interacts with discourse context during Chinese discourse reading. Event-related potentials were recorded while participants read two-sentence discourses, in which the first sentence provided the discourse context that either introduced a gender stereotype-countering attitude towards roles, such as “One should strive for the target job, and getting a job should not be restricted by gender”., or was neutral. The second sentence contained the critical clause in which the stereotypical gender of the object noun (a role name) was either consistent or inconsistent with the gender specified by the head noun (a kinship term) of the subject noun phrase, as in “Li’s [daughter/son] became a nurse…”. The object nouns elicited a larger N400 and a larger late negativity (LN) for the inconsistent compared to the consistent conditions in the neutral contexts. Crucially, when the discourse context offered information countering gender stereotypes, both the N400 and LN effects were reversed, with the negativities being smaller for the inconsistent compared to the consistent conditions. The reversal of the N400 effects suggests that discourse contexts can immediately override the processing of gender stereotypes, and thus readers compute discourse context and local pragmatic information simultaneously during discourse reading.

## 1. Introduction

When people read or listen to a sentence or discourse, they need not only to access its literal meaning on the basis of linguistic information such as grammatical category, morphosyntax, word order, and lexical semantics, but also to evaluate whether or not the literal meaning is consistent with their world knowledge (including social knowledge or beliefs such as gender stereotypes). For example, readers could experience processing difficulties while reading sentences like “Li’s son became a nurse when he left school”, as the stereotypical gender of “nurse” (nurses are stereotypically female) was inconsistent with the definitional gender of “son”, inducing a world knowledge anomaly. Within psycholinguistics and neurolinguistics, the use of world knowledge during language processes is often referred to as pragmatic processing (see [1] for a discussion about the distinction between the domains of semantics and pragmatics). In the present study, we focus on the pragmatic processing of social knowledge (gender stereotypes for role names), especially its interaction with the processing of discourse context during discourse reading to shed light on how social knowledge is used in language comprehension, an issue that has not been well addressed.

In the research field of discourse comprehension, a number of previous studies have examined how local sentence-level syntactic, semantic, and/or pragmatic processing interacts with the processing of discourse context (e.g., [2,3,4,5,6,7,8,9]). However, it is still not clear how local sentence-level pragmatic processing of social knowledge interacts with global discourse processing during discourse reading. In the present study, we investigated this issue by examining whether and how local sentence-level pragmatic processing of gender stereotypes for role names, as in “Li’s son became a nurse…”, is influenced by a discourse context that provides information countering gender stereotypes, such as “One should strive for the target job, and getting a job should not be restricted by gender”.

Using a plausibility violation paradigm, previous studies have documented inconsistent results regarding whether or not discourse context has an early influence on local sentence-level semantic and/or pragmatic processing (e.g., [2,3,4,5,6,8]). In some of these studies (e.g., [2,8]), discourse context did not influence initial sentence-level plausibility processing, though it did influence later processing of the local plausibility. For example, Warren et al. [8] found that as an early measure of eye movement reading, gaze durations at target words (e.g., “bread” in the examples below) were longer for sentences describing an impossible event via introducing a semantic feature mismatch between a verb and its argument (e.g., “Patricia/Harry used a book to teach the tough bread”) compared to their possible counterparts (e.g., “Patricia/Harry used a microwave to heat the tough bread”). Crucially, this early implausibility effect was not influenced by discourse context: fantasy contexts (e.g., a context talking about “Harry Potter”) did not affect the initial implausibility effect, although they did eliminate it later, as revealed by later measures (regression path times and total reading times) (see [2] for a similar role of counterfactual discourse contexts). The independence of initial local sentence processing from discourse context appears to be consistent with two-step models in which the local sentence-level computation temporally and/or functionally precedes the use of discourse context (e.g., [7,9,10,11,12,13,14]).

However, some other studies (e.g., [3,4,5,6,15,16]) have revealed an immediate influence of a variety of discourse contexts on the local sentence-level processing, which is consistent with one-step or interactive models, such as constraint-based models (e.g., [17,18]), the cue integration model [19], and the dynamic generative framework [20]. For these models, the discourse context and the information in a local sentence are permitted to be used simultaneously (see [21] for a review). For example, in an event-related brain potential (ERP) study, Filik and Leuthold [4] demonstrated that fictional discourse contexts can immediately make a locally pragmatically anomalous word (e.g., “lorry” in “He picked up the lorry…”) globally acceptable, resulting in the N400 effects for local pragmatic anomalies being immediately eliminated. In addition, the N400 effects for world knowledge violations in a local sentence (e.g., “The city Venice has many roundabouts…”) can be eliminated once discourse contexts make such violations more acceptable via shifting the relevant focus to support new information, such as “The large and increasing amount of cyclists in the inner city of Venice had to be regulated. The city council decided 10 years ago to replace traffic lights with other road layouts that ease traffic flow” [5]. Interestingly, Hald et al. [5] also found that even if discourse contexts made world knowledge violations in local sentences more acceptable, a larger N400 was still observed for such violations compared to the baseline condition in which both the discourse context and the local sentence meaning were consistent with world knowledge. These observations imply that discourse contexts play an immediate but limited role in modulating local pragmatic processing, at least under some circumstances.

As indicated by Warren et al. [8], the absence or presence of an early effect of discourse context on local plausibility processing could be due to, among others, the strength and/or types of local plausibility violations, which may influence the temporal point at which local plausibility violations are detected. The slower the local violations are detected, the more likely the discourse context shows an effect at the initial stage of local sentence processing. For example, as we have mentioned above, in Filik and Leuthold’s study [4], the local pragmatic anomalies were realized by violating world knowledge about the likely participants in events (e.g., a person picking up a lorry), which may be responsible for the relatively slow detection of local anomalies and, thus, the presence of an effect of discourse context on the initial processing of sentence-level plausibility. In contrast, in Warren et al.’s study [8], the local plausibility anomalies were realized by introducing a semantic feature mismatch between a verb and its argument (e.g., teaching a bread), which may bring about the relatively fast detection of local anomalies and, thus, the absence of an early or initial effect of discourse context.

The complex modes of the interplay between local sentence-level processing and the use of discourse context suggest that local sentence-level processing does not always temporally and/or functionally precede the processing of discourse context. Many factors, such as the strength and/or types of local plausibility violations, may influence the interplay mode. In the present study, we aimed to investigate how local sentence-level pragmatic processing of social knowledge interacts with the processing of discourse context. For this purpose, we examined whether the early processing of gender stereotypes for role names in a local sentence is influenced by discourse contexts that counter gender stereotypes. As we discuss below, stereotypical knowledge has been shown to be processed in a different way from typical lexical semantics [22]. However, as we have reviewed above, the mode of the interplay between stereotypical knowledge in local sentences and discourse contexts has not been sufficiently addressed in the literature, though there have already been a relatively large number of studies that examined how typical lexical semantics interplays with discourse contexts (e.g., [2,3,4,6,7,8,9,15,16]). It would thus be interesting to see how stereotypical knowledge in a local sentence interacts with discourse contexts.

So far, there has been evidence that gender stereotype information can be activated immediately and automatically when a role name (e.g., “nurse”) or an action verb (e.g., “cut”) is being read (e.g., [22,23,24,25,26,27,28,29,30,31,32,33,34]). In addition, there have been a few studies that examined how gender stereotype information interplays with other types of information [22,25,35]. One of these studies found a similar N400 response for all three types of inconsistencies between stereotypical gender of role names and grammatical gender cues in Spanish, i.e., stereotypical inconsistencies caused by morphologically anomalous noun ending, as in “las mineras” (‘the_[+F]_ female miners’), syntactic inconsistencies caused by anomalous gender marked determiner, as in “las mineros” (‘the_[+F]_ male miners’), and double inconsistencies caused by both morphologically anomalous noun ending and anomalous gender marked determiner, as in “los mineras” (‘the_[+M]_ female miners’) [22]. As Molinaro et al. [22] have indicated, this finding suggests that gender stereotype information can override grammatical gender morphology and thus there is a functional priority of stereotypical knowledge over syntactic cues. Given that the mode of the interplay between stereotypical knowledge and morphosyntax differs from that between lexical semantics and morphosyntax, Molinaro et al. [22] argued that stereotypical knowledge is processed differently from typical lexical semantics (see [36] for eye-tracking data showing a qualitative difference between the processing of stereotypical and definitional gender).

More relevant to the present study, discourse contexts have been shown to have an impact on the processing of gender stereotype information in local sentences [25,35]. Lassonda and O’Brien [35] have found such an influence. They observed longer reading time for a target sentence when the subject pronoun (“she”) of the sentence specified a gender that was inconsistent with the stereotypical gender for its referent, the target occupation character that is stereotypically male (e.g., “firefighter”), compared to the target sentence containing a consistent pronoun (“he”). Crucially, no inconsistency effects were observed when the preceding discourse context explicitly specified the gender of the target occupation character. The elimination of inconsistency effects does indicate an effect of discourse context on the processing of gender stereotype information in a local sentence. However, this finding cannot be used to address the detailed time course of the discourse context effect, as the measure of reading time was focused on the whole target sentence that began with the subject pronoun (“he” or “she”) and was unable to reflect a real-time interpretation of the target occupation character in gender-specifying discourse context.

Duffy and Keir [25] also manipulated both the discourse context and the gender consistency between the role name and reflexive pronoun. They found that gender stereotype inconsistencies, as in the target sentence “The electrician taught herself…”, resulted in a longer first pass and go-past times on the reflexive pronoun compared to their consistent counterparts in neutral discourse contexts, suggesting an immediate use of gender stereotype information during sentence comprehension. Crucially, this early effect of stereotype inconsistency was eliminated when preceding discourse contexts explicitly defined the gender of the role-named character, such as “The electrician was a cautious woman who carefully secured her ladder…”. However, given that the stereotype inconsistency effects were measured on the reflexive pronoun, the second word after the role name in the target sentence, it is not clear exactly when the gender-defining discourse context influenced the processing of gender stereotypes for the role names.

Indeed, as Duffy and Keir [25] have argued, the elimination of inconsistency effects is congruent with the lexical reinterpretation model proposed by Hess et al. [37]. According to this model, a continual updating of discourse representation can result in a word being reinterpreted in accordance with the global discourse model. For example, when the role name in the target sentence “The electrician taught herself…” was read, it could have been lexically reinterpreted as having a gender of female within the gender-defining discourse context “The electrician was a cautious woman who carefully secured her ladder…”. Therefore, there could be no genuine conflict of gender between the role name and the reflexive pronoun when the target sentence was being read, such that the measure of eye movements on the reflexive pronoun may not be able to reflect an early effect of discourse context on gender stereotype processing for the role names.

In the present study, we investigated the neural and temporal dynamics underlying the influence of discourse context on the processing of gender stereotype information in local sentences using the ERP technique. To overcome methodological limitations in previous studies [25,35] and to address exactly when discourse contexts influence the processing of stereotype consistencies in local sentences, we focused our online measure of stereotype consistency effects on role names in target sentences. We used a full factorial design that crossed two factors: context (neutral vs. stereotype-countering) and consistency (consistent vs. inconsistent). As illustrated in Table 1, the object noun (always a role name, e.g., “nurse”) in the critical clause served as the critical word (CW) in the ERP experiment. The stereotypical gender of the CW was either consistent or inconsistent with the gender specified by the head noun (always a kinship term, e.g., “daughter” or “son”) of the subject noun phrase. In addition, the first sentence of each short discourse (always containing two sentences) provided a context that either was neutral, as in (1) and (2), or contained gender stereotype-countering information via introducing gender-equality attitudes towards roles, as in (3) and (4).

We expected a larger N400 at the CW in an early time window for the NI compared to the NC conditions, as in Molinaro et al. [22] that also used role names as CWs for ERPs (A single P600 response has been observed for gender stereotype inconsistencies when a pronoun instead of a role name was used as the CW (e.g., [23,27,29,34]). It thus appears that the types of CWs (role names or pronouns), among others, could be relevant. We will return to this issue in the Section 4). The N400 could be followed either by a P600 or by a late negativity (LN) in the later time window, reflecting late processing of gender stereotype inconsistencies, as in previous studies (N400–P600: e.g., [30,32,38]; N400–LN: e.g., [22,31]).

More crucially, if the evaluation of gender stereotype consistencies in a local sentence temporally and/or functionally precedes the processing of discourse context, as assumed in two-step models (e.g., [7,9,10,11,12,13,14]), discourse contexts would influence later but not early ERP effects for stereotype consistencies, such that an interaction of consistency with context would be observed only in the later time window. We call this the Precedence Hypothesis. Alternatively, the processing of gender stereotypes for role names in local sentences can immediately interact with gender stereotype-countering discourse contexts that introduce gender-equality attitudes towards these roles. In that case, a reversed effect of gender stereotype consistencies in the early time window would be obtained, as that a local sentence in which a male engages in a stereotypically female role, and vice versa (e.g., (4) in Table 1), would be more congruent with such discourse contexts, compared to a local sentence containing a consistent role name, as in (3) in Table 1. We call this the Immediate Interaction Hypothesis, which is congruent with one-step models (e.g., [17,18,19,20]). In the present study, we test both hypotheses.

## 2. Methods

### 2.1. Participants

Forty-seven native Mandarin Chinese speakers gave informed consent to participate in the ERP experiment. They were paid for their participation. According to their own reports, all participants were right-handed, had normal or corrected-to-normal vision, and had no reading or neurological disorders. Data from three participants were excluded from the analyses, due to a small number of artifact-free trials (less than 75% of trials in any condition). Four participants quit due to being tired or sleepy after having finished 1 to 3 blocks (5 blocks total, see Section 2.3). Data from the remaining 40 participants (half females; mean ± S.D. age = 22.58 ± 2.68 years; age range = 18–29 years) were used in the statistical analyses. An analysis of variance (ANOVA) was used to provide a conservative estimate of the appropriate sample size, given that mixed-effects models, which we used for the analysis of ERP data (see Section 2.4), are arguably more sensitive. G*Power (version 3.1.9.7) [39] was used to calculate sample size. The minimum sample size of 36 has a power of 95% to detect a medium-sized effect (f = 0.25) at the significance level of 0.05. We decided to increase the sample size a little bit to optimize the statistical power. Thus, we aimed for 40 participants being included in the statistical analyses. This study was approved by the Human Subject Review Committee at Peking University.

### 2.2. Materials and Normative Measures

To create gender-stereotypically consistent or inconsistent sentences, 40 stereotypically male or female (half male-biased and half female-biased) role names were selected on the basis of a pretest of gender stereotypes for role names. In this pretest, a separate group of 48 participants (half females; mean ± S.D. age = 23.67 ± 2.20 years; age range = 18–28 years) was asked to judge the percentage of males or females in the population who engage in each particular role for 286 role names on an 11-point (0 to 100%) Likert scale based on their immediate intuitions. To maximize the effects of gender stereotype consistencies and to minimize the differences in the degree of gender stereotypes between the male-biased and female-biased role names, we selected 20 female-biased role names with the highest level of gender stereotypes and 20 male-biased role names that had a similar level of gender stereotypes to the female-biased ones. The mean (SD) percentage of males or females was 86.15% (12.01%) and 78.91% (14.23%) for male-biased and female-biased role names, respectively. In addition, 40 kinship names (half definitionally male, e.g., “son”, and half female, e.g., “daughter”) were used in order to realize gender-stereotypically consistent or inconsistent conditions.

Using these selected role and kinship names (each being used four times repeatedly), we created 160 sets of two-sentence short discourses that served as critical materials (see Table 1 for examples). The first sentence of the discourse provided either a neutral context (the NC and NI conditions), or a context that introduced gender equality or gender stereotype-countering attitudes towards roles (the SCC and SCI conditions). The second sentence contained three clauses, with the second one being the critical clause in which the stereotypical gender of the object noun (a role name) was either gender-stereotypically consistent (the NC and SCC conditions) or inconsistent with the gender defined by the head noun (a kinship name) of the subject noun phrase (the NI and SCI conditions). Within each set of critical items, the second sentence differed only in the kinship name (e.g., “daughter/son” in Table 1) between the consistent and inconsistent conditions for both neutral and stereotype-countering discourse contexts. For the whole sets of critical items in which male-biased and female-biased role names occurred equally frequently, the same set of kinship names was used for the consistent and inconsistent conditions.

The 160 sets of critical items were assigned to four experimental lists using a Latin square procedure. To offset the highly similar permutations of a small set of constructions and the very limited types of anomalies in the critical items (stereotype inconsistencies for the NI and SCI conditions and discourse context incongruences for the SCC condition), for each list, we mixed the 160 critical items with 200 filler discourses of various constructions (though also consisting of two sentences, as in critical items). Half of these filler items contained various other anomalies (referential ambiguities, semantic anomalies, or syntactic violations) that occurred in various word positions in a discourse.

To validate our manipulations of stereotype consistency and discourse context, we conducted two acceptability pretests, in which participants were asked to rate each critical clause (e.g., “Li’s daughter became a nurse, …” in Table 1) or discourse fragment (from the beginning of the discourses up to the critical words) for acceptability on an 11-point Likert scale, where 0 means “completely unacceptable” and 10 means “completely acceptable”. A separate group of 32 participants (half females; mean ± S.D. age = 22.16 ± 3.43 years; age range = 18–32 years) and another separate group of 32 participants (half females; mean ± S.D. age = 21.63 ± 2.89 years; age range = 18–28 years) participated in the clause and discourse fragment ratings, respectively. For the clause rating, the 160 sets of critical items were assigned to two experimental lists using a Latin square procedure. To offset the highly similar syntactic structures and the single anomalies (stereotype inconsistencies) in the critical items, for each list, we mixed the 160 critical items with 200 filler sentences of various constructions, half of which contained various syntactic and/or semantic anomalies. For the discourse fragment rating, the 160 sets of critical items were assigned to four experimental lists using a Latin square procedure. To offset the highly similar permutations of a small set of constructions and very limited types of anomalies in the critical items, for each list, we mixed the 160 critical items with 160 filler discourse fragments of various constructions. Half of these filler items contained various other anomalies (syntactic or semantic anomalies or referential ambiguities). For both ratings, to further counterbalance any potential order effects, for each list, a second version with a reverse order was created. Each participant received only one list.

Both acceptability ratings were analyzed with cumulative link mixed models using the clmm2 function in the ordinal package (version 2019.12-10) [40] in R (version 4.0.2) [41], which are appropriate for Likert scales [42]. The models began with a maximal random effect structure and were simplified by the backward stepwise method if they did not converge [43,44]. In addition, sum coding was used for the categorical factors [45]. For the clause rating, the maximal model revealed a main effect of consistency (β = 0.59, *SE* = 0.11, *z* = 5.21, *p* < 0.001) (see Table S1 for further details), with the clauses being rated less acceptable in the inconsistent (*M* = 8.38, *SD* = 2.55) than in the consistent conditions (*M* = 9.05, *SD* = 2.01), as expected.

For the discourse fragment rating, the interaction of consistency with context was removed from the random effects structure to obtain convergence. The models revealed main effects of both consistency (β = 0.55, *SE* = 0.10, *z* = 5.59, *p* < 0.001) and context (β = −0.67, *SE* = 0.10, *z* = −6.97, *p* < 0.001). In addition, there was an interaction between the two factors (β = 0.87, *SE* = 0.11, *z* = 7.76, *p* < 0.001) (see Table S2 for further details). Follow-up analyses with Bonferroni correction using the emmeans package (version 1.5.1) [46] showed that when the discourse contexts were neutral, the discourse fragments were rated less acceptable in the inconsistent (*M* = 6.92, *SD* = 2.89) compared to the consistent conditions (*M* = 7.30, *SD* = 2.70) (β = −0.41, *SE* = 0.15, *z* = −2.77, *p* = 0.01), as expected. In contrast, when the discourse contexts provided stereotype-countering information, the discourse fragments were rated more acceptable in the inconsistent (*M* = 7.59, *SD* = 2.51) compared to the consistent conditions (*M* = 4.14, *SD* = 3.05) (β = 3.09, *SE* = 0.39, *z* = 7.87, *p* < 0.001). This is also expected, given that it was in the inconsistent condition that the local sentence described a situation (a male engaging in a stereotypically female role, and vice versa) that was congruent with the stereotype-countering discourse context. These results above suggest that our manipulation of contexts was valid.

Given that the cloze probabilities for the critical words (CWs) may influence the ERPs of interest (e.g., N400 [47]), we performed a discourse completion test. In this test, a separate group of 48 participants (25 females; mean ± S.D. age = 22.31 ± 2.58 years; age range = 18–29 years) was presented with the discourse fragments up to but not including the CWs and was asked to continue each discourse fragment with the first word that came to mind. The 160 sets of critical fragments were assigned to four experimental lists using a Latin square procedure. To offset the highly similar permutations of a small set of constructions in the critical items, for each list, the 160 critical items were mixed with 160 filler discourse fragments of various constructions. To further counterbalance order effects, for each list, a second version with a reverse order was created. Each participant received only one list. The mean (SD) cloze probabilities for the CWs were 7.24% (25.92%), 5.10% (22.01%), 3.96% (19.50%), and 7.50% (26.34%) for the NC, NI, SCC, and SCI conditions, respectively. The cloze probability was included as a predictor in the ERP data analyses to statistically control for the influence of the cloze probabilities on the ERPs (see the Section 3).

### 2.3. Procedure

Participants were seated in a dimly lit and sound-attenuated room at an approximate distance of 100 cm from a computer screen. All stimuli were presented in white characters on a black background and in the center of the screen. Each trial started with an 800 ms fixation cross, followed by a 500 ms blank screen. Then the first sentence of the discourse was presented as a whole for 5500 ms, followed by an 800 ms blank screen. And then the first clause of the second sentence was presented for 500 ms, followed by a blank screen for another 500 ms. After this, the rest of the second sentence was presented segment by segment (word or short phrase) at a rate of 800 ms per segment (400 ms word or phrase, 400 ms blank screen). Participants were advised to try to avoid blinking during the presentation of the discourse stimuli in order to reduce eye movements during the critical epochs. After the last segment, the screen was blank for 800 ms, followed one fourth of the time by a yes/no comprehension question. Only coherent discourses (i.e., those in the NC and SCI conditions) were followed by a question. Participants were asked to read the discourses carefully and answer the yes/no comprehension questions by pressing one of two response keys (“D” or “K”) on a computer keyboard. The response keys were counterbalanced across participants. The questions remained on the screen until the participant had responded or for a maximum of 3 s. The next trial began after a 2-s interval.

Each participant received only one of the four experimental list. For each list, the total 360 short discourses were evenly divided into five blocks with a short break between blocks. Before the formal experiment, participants completed 20 practice trials.

Given that the target ERPs might be influenced by potential individual differences in the degree of gender stereotypes, after EEG recording, a sudden posttest was conducted to enable statistical evaluation of the effect of individualized gender stereotypes (on the levels of both participant and item) on the target ERPs. As in the pretest of gender stereotypes for role names, in this posttest, on an 11-point (0 to 100%) Likert scale, participants judged the percentage of males or females in the population who engage in each particular role for 60 role names (40 CWs used in the ERP experiment being pseudorandomly mixed with 20 filler role names that were stereotypically neutral according to the pretest for role names). These rating scores were treated as a predictor of the target ERPs in ERP data analyses. The whole experiment lasted about two and a half hours.

### 2.4. EEG Recording and ERP Data Analysis

The electroencephalogram (EEG) was recorded from 64 Ag/AgCl electrodes mounted in an elastic cap (EASYCAP GmbH, Worthsee-Etterschlag, Germany) and was amplified with BrainAmps amplifiers (Brain Products, Munich, Germany) with an online bandpass of 0.016–100 Hz at a sampling rate of 1 kHz. Recordings were online referenced to the nose tip. Horizontal and vertical electrooculogram (EOG) were recorded from electrodes at the outer canthus of each eye and above and below the right eye, respectively. Electrode impedances were kept below 5 kΩ.

Offline, the EEG was downsampled at 250 Hz, was re-referenced to the average of the left and right mastoids, and was band-pass filtered at 0.1–40 Hz using a zero-phase IIR Butterworth filter (24 dB/oct). Ocular artifacts were removed from the EEG signal using Independent Component Analysis (ICA) implemented in BrainVision Analyzer (v.2.0, Brain Products, Munich, Germany). The epoch length was 1200 ms, ranging from 200 ms before to 1000 ms after the CW onset. All epochs with amplitudes above ±75 μV were excluded automatically, resulting in the exclusion of 7.31% of all trials (NC: 7.37%; NI: 7.37%; SCC: 6.75%; and SCI: 7.75%).

A 200 ms pre-CW baseline correction was performed only for plotting purposes. For statistical analysis of ERP data, we did not perform a traditional baseline correction. Instead, in order to objectively (statistically) determine how much baseline correction was needed and to increase statistical power by reducing residual error, we analyzed single-trial ERP amplitudes with a linear mixed-effects model, in which the scaled trial-wise mean amplitude in the 200 ms pre-CW interval was included as a predictor for ERPs of interest, as suggested by Alday [48]. These analyses were performed using the lmer function in the packages lme4 (v.1.1–26) [49] and lmerTest (v.3.1–2) [50] for R (v.4.0.2) [41]. In addition, all continuous variables were z-transformed before analysis, and all categorical variables were coded using sum coding.

All models included the fixed effects for consistency, context, the mean voltage in the 200 ms pre-CW (baseline) interval, cloze probability, degree of stereotypes, and all interactions except for a two-way interaction between cloze probability and degree of stereotypes for model parsimony. The models began with the maximal random effect structure and were simplified by removing random effects with a backward stepwise procedure using the anova function until convergence [43,44]. We report both the best models and the corresponding estimates of the fixed effects. p values were derived by type II Wald chi-square tests using the car R package (v.3.0-9) [51]. When the interaction between consistency and context was significant, pairwise comparisons of estimated marginal means were performed between the consistent and inconsistent conditions at each level of context using the emmeans R package (v.1.5.1) [46]. In these cases, Bonferroni corrections were applied, and corrected *p*-values are reported.

## 3. Results

Participants had a high accuracy on the yes/no comprehension questions (*M* = 93.60%; *SD* = 3.70%), suggesting that they read the discourses attentively.

To determine the spatiotemporal patterns of the ERP effects for stereotype consistencies in neutral contexts, which would provide a basis for examining the influence of stereotype-countering context on the processing of stereotype consistencies, we performed a visual inspection of ERP waveform differences between the NI (neutral-inconsistent) and NC (neutral-consistent) conditions. Two time windows were chosen for statistical analysis: (a) 350–600 ms for N400 effects and (b) 800–1000 ms for late negativity (LN) effects, based on the visual inspection, and to minimize the temporal overlap between these two negativities. In addition, we chose eight widely distributed electrodes (FC1, FC3, FC5, Cz, Pz, P4, P6, and O1) at which the N400 effects for stereotype inconsistencies were most pronounced, and eight left-posterior electrodes (CP3, CP5, P3, P5, P7, PO3, PO7, and O1) at which the LN effects were most pronounced.

**Figure 1:**
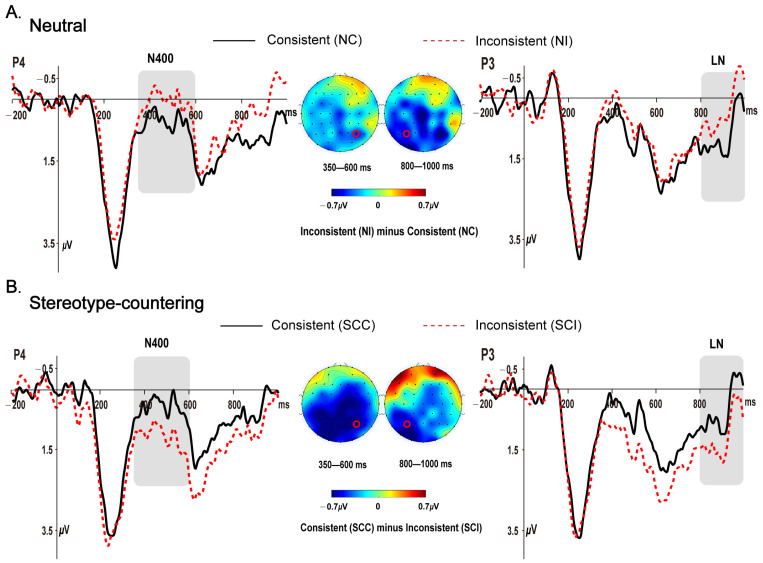
ERPs time locked to the onset of the critical words. Grand average ERPs for the consistent and inconsistent conditions at electrodes P4 (left side of the figure) and P3 (right side of the figure) in the neutral (**A**) and stereotype-countering discourse contexts (**B**), respectively, together with the scalp topographies of the corresponding difference waves (left and right red circles indicate electrodes P4 and P3, respectively; note that the difference waves in (**B**) were obtained by subtracting the inconsistent from the consistent conditions).

Figure 1A,B show the grand average ERPs elicited by the CWs (role names) for all four conditions (NC, NI, SCC, and SCI), and the scalp topographies of the difference waves of NI minus NC and SCC minus SCI for the neutral and stereotype-countering discourse contexts, respectively, in the two time windows. As shown in Figure 1A, the CWs elicited a larger N400 and LN for the inconsistent compared to the consistent conditions in the neutral discourse contexts. In contrast, the typical consistency effects were reversed by the stereotype-countering discourse contexts, with both the N400 and LN being smaller for the inconsistent compared to the consistent conditions, as shown in Figure 1B. The reversed consistency effects were expected for stereotype-countering discourse contexts, given that it was in the inconsistent instead of consistent condition that the local sentence was congruent with stereotype-countering discourse contexts. These visual impressions were statistically confirmed by the analysis using mixed-effects models.

Table 2 and Table 3 show the estimates of the fixed effects from the mixed-effects models for the 350–600-ms and 800–1000-ms time windows, respectively. Table 4 shows the results of an analysis of deviance (Type II Wald chi-square tests) using mixed-effects models in the 350–600-ms and 800–1000-ms time windows, respectively.

In the N400 (350–600 ms) time window, the random effects included by-participant and by-item intercepts and slopes for context (The random effects of items were considered for both the N400 and the LN time windows, due to potential between-item differences in ERP amplitudes for both time windows. Note that the trial-wise mean amplitude in the 200 ms baseline interval was included as a predictor for ERPs of interest, due to its potential systematic influence on ERP amplitudes in the later (N400 and LN) time windows. This does not create a conflict of principles, given that different aspects of trial-wise mean amplitudes were considered for the fixed and random effects of items, respectively). As shown in Table 4, there was an interaction of consistency with context. Post-hoc pairwise comparisons of estimated marginal means showed that when the discourse contexts were neutral, the CWs (role names) elicited a larger N400 in the inconsistent (*M* = 1.320 μV, *SE* = 0.396, 95% CI [0.529, 2.120]) than in the consistent conditions (*M* = 1.910 μV, *SE* = 0.396, 95% CI [1.112, 2.700]), β = −0.079, *SE* = 0.035, 95% CI [−0.147, −0.010], *t* (5606) = −2.257, *p* = 0.048. This is the expected consistency effect, reflecting an early processing of gender stereotypes when the role names were being read. Crucially, when the discourse contexts provided information countering gender stereotypes, the consistency effects were reversed, with the N400 being smaller for the inconsistent (*M* = 2.330 μV, *SE* = 0.416, 95% CI [1.495, 3.160]) than for the consistent conditions (*M* = 1.740 μV, *SE* = 0.416, 95% CI [0.903, 2.570]), β = 0.080, *SE* = 0.035, 95% CI [0.011, 0.149], *t* (5674) = 2.267, *p* = 0.046. The reversal of the consistency effects should be expected if the stereotype-countering discourse contexts can immediately influence the processing of gender stereotypes in a local sentence.

In the LN (800–1000 ms) time window, the random effects included random intercepts for both participants and items and by-participant random slopes for context. As shown in Table 4, there was an interaction of consistency with context. Post-hoc pairwise comparisons of estimated marginal means showed that in the neutral discourse contexts, the CWs elicited a larger LN for the inconsistent (*M* = 0.413 μV, *SE* = 0.336, 95% CI [−0.258, 1.080]) compared to the consistent conditions (*M* = 1.101 μV, *SE* = 0.335, 95% CI [0.431, 1.770]), β = −0.081, *SE* = 0.036, 95% CI [−0.152, −0.011], *t* (5731) = −2.255, *p* = 0.048. The LN effects may reflect the late processing (a reprocessing and/or a repair) of gender stereotypes inconsistencies. Similar to the N400 effects, the LN effects obtained in the neutral contexts were reversed by the stereotype-countering discourse contexts: the LN was smaller for the inconsistent (*M* = 0.506 μV, *SE* = 0.347, 95% CI [−0.188, 1.200]) compared to the consistent conditions (*M* = 1.568 μV, *SE* = 0.346, 95% CI [0.877, 2.260]), β = 0.126, *SE* = 0.036, 95% CI [0.054, 0.197], *t* (5769) = 3.456; *p* = 0.001. The reversal of the LN effects suggests a benefit to the inconsistent condition from the stereotype-countering contexts in the later time window.

In sum, we found both a larger N400 and a larger LN for the inconsistent compared to the consistent conditions in the neutral discourse contexts. More importantly, both effects were reversed by the stereotype-countering discourse contexts.

## 4. Discussion

The goal of the present study is to investigate whether or not discourse contexts influence the early processing of gender stereotypes for role names in a local sentence. For this purpose, we manipulated both discourse context and gender stereotype consistency for role names, such that in both neutral and stereotype-countering discourse contexts, the stereotypical gender of the role name was either consistent or inconsistent with the definitional gender of the kinship term in the local sentence. When the discourse contexts were neutral, compared with the consistent role names, the inconsistent ones elicited a larger N400 and a larger LN in the 350–600 ms and 800–1000 ms time windows, respectively, as in some previous studies (e.g., [22,31]). The N400 responses may reflect an early detection of gender mismatches and/or difficulty in retrieving or integrating lexical information due to gender mismatches for the inconsistent role names. The LN effects may reflect a repair or resolution of gender mismatches for the inconsistent condition, probably via suppressing the prevalent gender stereotype and switching to an alternative interpretation for role names (e.g., “a nurse can also be a male, though not very often”). Interestingly, the LN effects have also been observed for the processing of syntactic and/or semantic anomalies (e.g., [52]) and other types of pragmatic anomalies such as construction-based pragmatic violations of event likelihood [53], scalar implicature violations [54] and pragmatic violations of social status information [55], among others. It is thus possible that the LN effect reflects a reinterpretation process that is necessary in the face of a variety of anomalies during sentence or discourse comprehension.

As we have noted in the Introduction section, in some previous studies, gender stereotype inconsistencies have been shown to be also associated with either a single P600 (e.g., [23,27,29,34]) or a biphasic N400-P600 response (e.g., [30,32,38]). The presence or absence of a P600 for gender stereotype inconsistencies could be either related to the types of critical words for ERPs or due to individual differences, among others. For example, the studies that used a pronoun instead of a role name as the critical words have consistently observed a P600 (e.g., [23,29,32,34]). In addition, it is possible that some participants might process gender mismatches only for the purpose of monitoring processing errors, resulting in a P600 response for such anomalies (see [7,9] for the discussion of P600-related individual difference in processing discourse referential ambiguities). Despite its importance, the potential factors that result in the inconsistent ERP effects for gender stereotype processing are beyond the goal of the present study. We look forward to future research addressing this issue.

More importantly, when the discourse contexts introduced a gender-equality attitude towards roles and thus provided information countering gender stereotypes, the typical gender consistency effects were reversed, that is, both the N400 and LN were smaller for the inconsistent compared to the consistent conditions. Note that it was in the inconsistent instead of consistent condition that the local sentence described a situation (a male engaging in a stereotypically female role, and vice versa) that was congruent with stereotype-countering discourse contexts. The reversal of the consistency effects was therefore expected.

As we have indicated in the Introduction section, for some previous studies (e.g., [25,35]), the measure of stereotype inconsistency effects cannot definitely reflect a real-time or early influence of discourse context on the processing of gender stereotypes for a role name. These studies did not online measure the processing of role names in target sentences; instead, the reading time was measured either for whole target sentences [35] or for reflexive pronouns [25]. In contrast, in the present study, we online measured the processing of role names in a specific discourse context. Therefore, the discourse context effects we observed—the consistency effects obtained in the neutral context being reversed by the stereotype-countering contexts—can reflect a real-time influence of discourse context on the interpretation of gender stereotype information in local sentences.

The reversal of N400 effects for gender inconsistencies appears to be congruent with the Immediate Interaction Hypothesis, which we have proposed on the basis of one-step models that permit the simultaneous use of discourse context and the information in local sentences (e.g., [17,18,19,20]; see [21] for a review). According to this hypothesis, the information of gender stereotypes for role names in a local sentence can immediately interact with discourse contexts, such that the stereotype-countering discourse contexts can immediately make easier the interpretation of role names in the inconsistent compared to the consistent conditions, resulting in a reversed N400 effect.

Our observation of an early discourse context effect is obviously inconsistent with the Precedence Hypothesis or two-step models in which local sentence-level computations are temporally and/or functionally prior to the processing of discourse context (e.g., [7,9,10,11,12,13,14]). According to this type of account, the processing of gender stereotype consistencies in local sentences temporally and/or functionally precedes the use of discourse context, such that discourse contexts would influence later but not early ERP effects for stereotype consistencies. However, this is not the case. The results of this study, together with those of previous studies documenting an immediate influence of discourse contexts on local pragmatic processing (e.g., [3,4,5,6]), suggest that local sentence-level pragmatic processing of both social and non-social knowledge can immediately interact with discourse context processing. Thus, although there has been evidence for a functional priority of local (syntactic and/or semantic) phrase-structure processing over the processing of discourse referential contexts [7,9], local sentence-level computations do not always necessarily precede the processing of discourse contexts.

The complex modes of the interplay between local sentence-level processing and the use of discourse context may be related to the relative weight of sentence-level and discourse level processing. For example, compared with the processing of discourse referential ambiguities, the processing of local syntactic and/or semantic phrase-structure anomalies could be weighted to a greater extent, resulting in the former being blocked by the latter [7,9]. The participants in Hald et al.’s ERP study [5], which we have discussed earlier, could probably weight the processing of discourse contexts and world knowledge in local sentences to a similar extent, such that both discourse context anomalies and world knowledge anomalies resulted in an increase in N400 amplitude at the critical word. For the participants in our study, the processing of stereotype-countering discourse contexts could probably be weighted to a greater extent than the computation of local gender stereotype consistencies, which caused the reversal of the gender consistency effects. We look to future research to explore these speculations.

## 5. Conclusions

To conclude, our results show that stereotype-countering discourse contexts can immediately influence the local sentence-level pragmatic processing of gender stereotypes, an important type of social knowledge. To further get an insight into how the brain weights different types of pragmatic cues related to social knowledge relative to discourse-level cues, future research may examine the neurocognitive dynamics underlying how other types of social knowledge in a local sentence interplay with different types of discourse contexts.

## Figures and Tables

**Table 1:** Design and discourse examples for all four critical conditions. Examples are given in Chinese, with English glosses and translations. The critical words are in bold.

Condition	Example
Neutral, consistent (NC)	(1) 喜欢一份工作就应该努力争取，不要被困难吓倒。/一番努力后，/老李的/女儿/成为了/一名/护士，/现在/工作得/得心/应手。
	One should strive for the target job, and should not be afraid of difficulties./After much effort,/Li’s/daughter/became/a/nurse,/and now/works very well.
Neutral, inconsistent (NI)	(2) 喜欢一份工作就应该努力争取，不要被困难吓倒。/一番努力后，/老李的/儿子/成为了/一名/护士，/现在/工作得/得心/应手。
	One should strive for the target job, and should not be afraid of difficulties./After much effort,/Li’s/son/became/a/nurse,/and now/works very well.
Stereotype-countering, consistent (SCC)	(3) 喜欢一份工作就应该努力争取，不要被性别限制。/一番努力后，/老李的/女儿/成为了/一名/护士，/现在/工作得/得心/应手。
	One should strive for the target job, and getting a job should not be restricted by gender./After much effort,/Li’s/daughter/became/a/nurse,/and now/works very well.
Stereotype-countering, inconsistent (SCI)	(4) 喜欢一份工作就应该努力争取，不要被性别限制。/一番努力后，/老李的/儿子/成为了/一名/护士，/现在/工作得/得心/应手。
	One should strive for the target job, and getting a job should not be restricted by gender./After much effort,/Li’s/son/became/a/nurse,/and now/works very well.

**Table 2:** Estimates of fixed effects in the N400 (350–600 ms) time window.

	β	*SE*	*df*	95% CI	*t*	*p*
intercept	−0.007	0.050	43.41	[−0.11, 0.09]	−0.15	0.881
baseline	0.009	0.012	5842	[−0.02, 0.03]	0.72	0.470
cloze	0.010	0.015	373.2	[−0.02, 0.04]	0.79	0.433
consistency	0.000	0.012	5649	[−0.02, 0.02]	−0.02	0.982
context	−0.030	0.015	37.23	[−0.06, 0.00]	−1.94	0.060
degree of stereotypes	−0.020	0.014	3457	[−0.05, 0.01]	−1.53	0.126
baseline:cloze	0.004	0.012	5848	[−0.02, 0.03]	0.26	0.799
baseline:consistency	0.020	0.012	5852	[−0.01, 0.04]	1.16	0.244
baseline:context	−0.005	0.012	5854	[−0.03, 0.02]	−0.40	0.689
baseline:degree of stereotypes	0.016	0.012	5849	[−0.01, 0.04]	1.32	0.186
cloze:consistency	0.014	0.013	5491	[−0.01, 0.04]	1.06	0.289
cloze:context	−0.001	0.013	281.6	[−0.03, 0.02]	−0.11	0.915
consistency:context	0.040	0.012	5669	[0.02, 0.06]	3.20	0.001
consistency:degree of stereotypes	0.020	0.012	5776	[−0.01, 0.04]	1.59	0.113
context:degree of stereotypes	0.020	0.013	1633	[0.00, 0.04]	1.29	0.198
baseline:cloze:consistency	0.000	0.012	5858	[−0.02, 0.02]	0.03	0.974
baseline:cloze:context	0.026	0.012	5841	[0.002, 0.05]	2.16	0.031
baseline:consistency:context	−0.004	0.012	5849	[−0.03, 0.02]	−0.33	0.740
baseline:consistency:degree of stereotypes	−0.013	0.012	5851	[−0.04, 0.01]	−1.08	0.281
baseline:context:degree of stereotypes	0.007	0.012	5864	[−0.02, 0.03]	0.52	0.602
cloze:consistency:context	0.003	0.013	5425	[−0.02, 0.03]	0.24	0.809
consistency:context:degree of stereotypes	0.012	0.012	5771	[−0.01, 0.04]	0.95	0.344
baseline:cloze:consistency:context	−0.008	0.012	5838	[−0.03, 0.02]	−0.63	0.529
baseline:consistency:context:degree of stereotypes	−0.021	0.012	5843	[−0.05, 0.00]	−1.69	0.092

**Table 3:** Estimates of fixed effects in the LN (800–1000 ms) time window.

	β	*SE*	*df*	95% CI	*t*	*p*
intercept	−0.002	0.033	40.42	[−0.07, 0.07]	−0.04	0.965
baseline	−0.030	0.013	5893	[−0.06, 0.00]	−2.30	0.022
cloze	0.012	0.013	308.1	[−0.01, 0.04]	0.90	0.371
consistency	−0.011	0.013	5719	[−0.04, 0.01]	−0.86	0.389
context	−0.017	0.014	40.32	[−0.04, 0.01]	−1.18	0.243
degree of stereotypes	−0.010	0.014	2261	[−0.04, 0.02]	−0.74	0.459
baseline:cloze	−0.010	0.014	5882	[−0.04, 0.02]	−0.70	0.487
baseline:consistency	−0.015	0.013	5878	[−0.04, 0.01]	−1.12	0.261
baseline:context	−0.013	0.013	5887	[−0.04, 0.01]	−0.98	0.328
baseline:degree of stereotypes	0.007	0.013	5888	[−0.02, 0.03]	0.52	0.600
cloze:consistency	0.003	0.013	5576	[−0.02, 0.03]	0.24	0.812
cloze:context	0.002	0.013	4131	[−0.02, 0.03]	0.12	0.905
consistency:context	0.052	0.013	5779	[0.03, 0.08]	4.04	<0.001
consistency:degree of stereotypes	0.005	0.013	5850	[−0.02, 0.03]	0.41	0.680
context:degree of stereotypes	0.013	0.013	2405	[−0.01, 0.04]	0.98	0.326
baseline:cloze:consistency	0.011	0.014	5868	[−0.02, 0.04]	0.84	0.402
baseline:cloze:context	0.016	0.014	5885	[−0.01, 0.04]	1.17	0.242
baseline:consistency:context	0.025	0.013	5884	[0.00, 0.05]	1.96	0.050
baseline:consistency:degree of stereotypes	0.026	0.013	5891	[0.00, 0.05]	1.98	0.048
baseline:context:degree of stereotypes	0.021	0.013	5885	[−0.01, 0.05]	1.58	0.113
cloze:consistency:context	−0.001	0.013	5574	[−0.03, 0.03]	−0.04	0.970
consistency:context:degree of stereotypes	0.011	0.013	5840	[−0.01, 0.04]	0.82	0.410
baseline:cloze:consistency:context	0.020	0.014	5896	[−0.01, 0.05]	1.45	0.147
baseline:consistency:context:degree of stereotypes	−0.011	0.013	5890	[−0.04, 0.01]	−0.84	0.400

**Table 4:** Analysis of deviance (Type II Wald chi-square tests) for the N400 (350–600 ms) and LN (800–1000 ms) time windows.

	Df	N400		LN	
		χ^2^	*p* (>χ^2^)	χ^2^	*p* (>χ^2^)
baseline	1	0.669	0.413	4.186	0.041
cloze	1	0.744	0.388	0.826	0.363
consistency	1	0.002	0.967	0.576	0.448
context	1	3.544	0.060	1.472	0.225
degree of stereotypes	1	2.439	0.118	0.599	0.439
baseline:cloze	1	0.128	0.720	0.180	0.671
baseline:consistency	1	1.494	0.222	1.184	0.277
baseline:context	1	0.208	0.649	0.979	0.322
baseline:degree of stereotypes	1	1.560	0.212	0.398	0.528
cloze:consistency	1	1.126	0.289	0.122	0.727
cloze:context	1	0.003	0.957	0.002	0.962
consistency:context	1	10.002	0.002	16.218	<0.001
consistency:degree of stereotypes	1	2.636	0.104	0.166	0.684
context:degree of stereotypes	1	1.612	0.204	0.939	0.333
baseline:cloze:consistency	1	0.000	0.999	0.930	0.335
baseline:cloze:context	1	4.486	0.034	1.282	0.258
baseline:consistency:context	1	0.115	0.734	3.466	0.063
baseline:consistency:degree of stereotypes	1	1.302	0.254	4.002	0.045
baseline:context:degree of stereotypes	1	0.242	0.623	2.348	0.125
cloze:consistency:context	1	0.051	0.821	0.002	0.964
consistency:context:degree of stereotypes	1	0.973	0.324	0.680	0.410
baseline:cloze:consistency:context	1	0.396	0.529	2.107	0.147
baseline:consistency:context:degree of stereotypes	1	2.840	0.092	0.713	0.399

## Data Availability

The data presented in this study are available on request from the corresponding author.

## Supplementary Materials

The following supporting information can be downloaded at: https://www.mdpi.com/article/10.3390/brainsci13030387/s1, Table S1: Summary of cumulative link mixed model fitted with the Laplace approximation for the rating scores in the pretest of clause acceptability; Table S2: Summary of cumulative link mixed model fitted with the Laplace approximation for the rating scores in the pretest of discourse fragment acceptability.
