# Supplementary material for: Discourse Context Immediately Overrides Gender Stereotypes during Discourse Reading: Evidence from ERPs
Source: Brain Sci. 2023 Feb 23;13(3):387. doi: 10.3390/brainsci13030387 (PMC10046660; doi:10.3390/brainsci13030387)
Supplement: Supplementary file 1 [file brainsci-13-00387-s001.zip › brainsci-2203232-supplementary.pdf]

Supplementary Materials

Table S1: Summary of cumulative link mixed model fitted with the Laplace approximation for the rating scores in the pretest of clause acceptability. Cumulative Link Mixed Model fitted with the Laplace approximation formula : `clmm (formula = rating ~ consistency + (consistency | sub) + (consistency | item), data = dis.pretest1.data`

link	threshold	nobs	logLik	AIC	niter	max.grad	cond.H
logit	flexible	5110	-5732.47	11498.93	2717(23545)	4.07e-03	2.4e+03

Random effects:

Groups	Name	Variance	Std.Dev.	Corr
item	(Intercept)	1.9801	1.4075	
	consistency[consistent]	0.1972	0.4441	-0.094
sub	(Intercept)	2.8350	1.6837	
	consistency[consistent]	0.3190	0.5648	0.026

Number of groups: item 160, subject 32

Coefficients:

	Estimate	Std. Error	z value	Pr(>|z|)
consistency[consistent]	0.5932	0.1139	5.207	1.92e-07 $***$

Signif. codes: 0 ‘$***$’ 0.001 ‘$**$’ 0.01 ‘$*$’ 0.05 ‘.’ 0.1 ‘’ 1

Threshold coefficients:

	Estimate	Std. Error	z value
0|1	-6.5277	0.3496	-18.669
1|2	-5.8537	0.3413	-17.151
2|3	-5.3241	0.3361	-15.840
3|4	-4.7659	0.3319	-14.359
4|5	-4.4929	0.3302	-13.605
5|6	-4.1136	0.3283	-12.529
6|7	-3.6870	0.3266	-11.289
7|8	-2.8480	0.3241	-8.787
8|9	-1.8924	0.3223	-5.872
9|10	-0.5484	0.3209	-1.709

Table S2: Summary of cumulative link mixed model fitted with the Laplace approximation for the rating scores in the pretest of discourse fragment acceptability. Cumulative Link Mixed Model fitted with the Laplace approximation formula : `clmm (formula = rating ~consistency * context + (consistency * context | sub) + (consistency * context | item), data = dis.pretest2.data`

link	threshold	nobs	logLik	AIC	niter	max.grad	cond.H
logit	flexible	5116	-9936.31	19938.62	9706(50636)	5.28e-03	2.3e+03

Random effects:

Groups	Name	Variance	Std.Dev.	Corr		
item	(Intercept)	0.19171	0.4379			
	consistency[consistent]	0.07413	0.2723	-0.296		
	context[neutral]	0.08386	0.2896	0.771	0.278	
	consistency[consistent]:context[neutral]	0.06078	0.2465	0.070	-0.618	-0.276
sub	(Intercept)	1.50627	1.2273			
	consistency[consistent]	0.25609	0.5061	-0.261		
	context[neutral]	0.26853	0.5182	0.422	-0.692	
	consistency[consistent]:context[neutral]	0.36646	0.6054	0.165	-0.828	0.837

Number of groups: item 160, subject 32

Coefficients:

	Estimate	Std. Error	z value	Pr(>|z|)
consistency[consistent]	-0.66950	0.09613	-6.965	3.29e-12 $***$
context[neutral]	0.54981	0.09830	5.593	2.23e-08 $***$
consistency[consistent]:context[neutral]	0.87280	0.11249	7.759	8.56e-15 $***$

Signif. codes: 0 '$***$' 0.001 '$**$' 0.01 '$*$' 0.05 '.' 0.1 ' ' 1

Threshold coefficients:

	Estimate	Std. Error	z value
0|1	-4.5418	0.2371	-19.160
1|2	-3.4049	0.2296	-14.832
2|3	-2.6898	0.2266	-11.869
3|4	-2.1765	0.2251	-9.667
4|5	-1.7014	0.2242	-7.590
5|6	-1.1591	0.2234	-5.189
6|7	-0.6348	0.2230	-2.847
7|8	0.1338	0.2227	0.601
8|9	1.0955	0.2232	4.909
9|10	2.3425	0.2252	10.401
